# Chinese International Students in the United States: The Interplay of Students’ Acculturative Stress, Academic Standing, and Quality of Life

**DOI:** 10.3389/fpsyg.2021.625863

**Published:** 2021-08-31

**Authors:** Zhaohui Su, Dean McDonnell, Feng Shi, Bin Liang, Xiaoshan Li, Jun Wen, Yuyang Cai, Yu-Tao Xiang, Ling Yang

**Affiliations:** ^1^Department of Geriatrics, Xinhua Hospital, Shanghai Jiao Tong University School of Medicine, Shanghai, China; ^2^Center on Smart and Connected Health Technologies, Mays Cancer Center, School of Nursing, UT Health San Antonio, San Antonio, TX, United States; ^3^Department of Humanities, Institute of Technology Carlow, Carlow, Ireland; ^4^Department of Research and Development, Shanghai United Imaging Intelligence, Shanghai, China; ^5^Department of Radiation Oncology, National Cancer Center/National Clinical Research Center for Cancer/Cancer Hospital, Chinese Academy of Medical Sciences and Peking Union Medical College, Beijing, China; ^6^Program of Public Relations and Advertising, Beijing Normal University-Hong Kong Baptist University United International College, Zhuhai, China; ^7^School of Business and Law, Edith Cowan University, Perth, WA, Australia; ^8^Shanghai Jiao Tong University School of Medicine, School of Public Health, Shanghai Jiao Tong University, China Institute for Urban Governance, Shanghai, China; ^9^Unit of Psychiatry, Department of Public Health and Medicinal Administration, Institute of Translational Medicine, Faculty of Health Sciences, Centre for Cognitive and Brain Sciences, Institute of Advanced Studies in Humanities and Social Sciences, University of Macau, Zhuhai, China

**Keywords:** acculturative stress, international students, school classification, quality of life, acculturation

## Abstract

**Background:**

Acculturation could cause grave health consequences in international students. However, there is a shortage of research into how acculturative stress might affect international students’ quality of life in light of their academic standing and experience. The lack of research is particularly pronounced among Chinese international students, representing the largest body of international students studying in the United States (U.S.). Thus, to bridge the research gap, this study aims to examine the interplay between international students’ acculturative stress, academic standing, and quality of life among a nationally representative sample of Chinese international students studying in the United States.

**Methods:**

An online survey that gauges Chinese international students’ levels of acculturative stress, academic standing, and quality of life was developed. Over 350 higher education institutions across the United States were approached, including public universities, private universities, and community colleges, among which approximately 220 institutions responded positively and supported survey distribution. A total of 751 students completed the survey. Multiple regression analyses were carried out to examine the associations between students’ acculturative stress, academic standing, and quality of life.

**Results:**

Findings reveal that acculturative stress negatively affects all four domains of Chinese international students’ quality of life, irrespective of their academic standing. Data analyses also show that compared to master’s and doctoral students, undergraduates experience the highest levels of acculturative stress. Furthermore, a significant difference emerged among undergraduate and doctoral international students’ acculturative stress levels, but not among undergraduate and master’s students, or master’s and doctoral students.

**Conclusion:**

Our study found that, compared to master’s and doctoral students, undergraduates had more significant acculturative stress associated with lower levels of quality of life. This finding highlights the potentially positive role of academic experience – while acculturative stress deteriorates international students’ quality of life, students’ academic standing and experience could be the protective factor in the equation. Future research could further examine how universities and colleges can capitalize on their academic apparatuses and resources to improve international students’ academic performance and students’ acculturation experience and quality of life.

## Background

Acculturative stress could be understood as the “reduction in the health status of the individuals who have to struggle to adjust to a new culture psychologically and socially” (Berry et al., 1987). Acculturative stress is unique because it occurs as a direct result of acculturation – the process and experience of people from differing cultural and/or social backgrounds interact with each other (Berry, 1980). While several studies suggest that the acculturation process has the potential to promote personal growth (e.g., multiculturalism) (Pedersen, 1991; Berry, 2005), for international students, due to the lack of adequate systematic support, acculturation often results in more harm than good concerning their physical and psychological health (Cho, 2003; Ye, 2006; Poyrazli and Lopez, 2007; Zhang and Goodson, 2011; Servaty-Seib et al., 2016). Existing literature on the impacts of acculturative stress on international students’ wellbeing, for instance, shows that students who experience acculturative stress encounter considerable adjustment issues, such as diminished sense of social belonging and pronounced levels of anxiety (Brunsting et al., 2018). Furthermore, research also shows that international students with high acculturative stress often exhibit severe psychological health issues, ranging from depression symptoms to suicidal ideation (Cho, 2003; Ye, 2006; Servaty-Seib et al., 2016).

However, while useful insights are available, little is known about how acculturative stress affects international students’ quality of life or how students’ academic standing and experience might be a protective factor against their acculturative stress. Quality of life refers to an “individuals’ perceptions of their position in life in the context of the culture and value systems in which they live, and in relation to their goals, expectations, standards, and concerns” (The WHOQOL, 1995). Compared to health outcomes like stress, depression, and suicidal ideation, quality of life is a more comprehensive concept that represents individuals’ overall sense of well-being (Bowling, 1991; Brazier et al., 1992; Nussbaum and Sen, 1993; Felce and Perry, 1995; The WHOQOL, 1995), which could provide a more balanced, connected, and complete picture of international students’ acculturation experiences.

It is also important to understand that academic standing and experience could play an essential role in shaping international students’ perception of acculturative stress. For instance, in a comparative study, researchers found that newly arrived international students have greater difficulty adjusting to living and academic environments than returning students (Poyrazli and Grahame, 2007). Compared with undergraduates, findings also show that graduate students generally have greater academic exposure and academic experience, and in turn, might be more skillful in coping with stressful situations, such as collaborating with students from diverse cultural backgrounds (Oswalt and Wyatt, 2014; Zvauya et al., 2017; Yusufov et al., 2019). However, due to a lack of research, little is known about how academic standing might shape the relationship between acculturative stress and quality of life among international students. To bridge the research gap, this study aims to examine the interplay between international students’ acculturative stress, academic standing, and quality of life in a nationally representative sample of Chinese international students studying in the United States. Chinese international students are used as a research sample as they are the largest body of international students in America. Specifically, we set out to address the following research questions:

• How does acculturative stress affect Chinese international students’ quality of life in light of their academic standing?
• How does academic standing influence the relationship between Chinese international students’ acculturative stress and quality of life?

## Materials and Methods

### Participants and Procedure

Chinese international students studying in the United States were invited to participate in this research from February to April, 2015. Participation requests were sent to International Student Offices and Chinese Students and Scholars Associations first, which were requested to help distribute the survey to their Chinese international students. Potential universities were selected via two approaches: (1) Based on Chinese Students and Scholars Associations that were present in the United States at the time (Immigration Road, 2015), which consisted mostly medium to large institutions arranged by states, and (2) purposeful and manually contacting universities and (community) colleges (e.g., U.S. News, 2021) that have enrolled Chinese international students but did not have a Chinese Students and Scholars Association and/or International Student Office presence. While over 350 institutions across the United States were approached, only approximately 220 universities’ International student offices and Chinese Students and Scholars Associations helped distribute the survey.

The authors’ university Institutional Review Board granted ethical approval for carrying out this research (IRB protocol number: 2014-12-0027). Students received an email explaining the purpose of the study and indicated that the completion and submission of the survey implied consent to participate. As an incentive, participants were entered into a draw for a $50 Amazon gift. In terms of eligibility, only participants who self-identified as Chinese international students enrolled in a higher education institution in the United States were invited to participate. Respondents were informed that they could withdraw from the study without penalty at any stage of the study. No force response mechanisms were employed as a measure to reduce potential psychological discomfort participants might experience.

### Study Measures

#### Sociodemographic Variables

Respondents provide information about their gender, age, marital status, academic standing, length of stay in the United States, and self-rated English proficiency. Length of stay and English proficiency were included in the study as previous research shows that they are significant predictors of international student acculturation experience (Williams and Berry, 1991; Lee et al., 2004; Smith and Khawaja, 2011; Lopez and Bui, 2014).

#### Acculturative Stress

Acculturative stress for international students was measured using a 36-item scale developed by Sandhu and Asrabadi (1994). An example item is, “people show hatred toward me non-verbally” (Sandhu and Asrabadi, 1994). Items were scored on a 5-point Likert-type scale (1 = strongly disagree, 5 = strongly agree). The total score for acculturative stress ranged from 36 to 180, with higher scores indicating greater acculturative stress. In this study, the Cronbach alpha for the total acculturative stress score was 0.96. Here, Cronbach alpha measures the score’s reliability ranging from 0 to 1, where a higher value means greater reliability.

#### Quality of Life

Quality of life was evaluated based on a four-domain structure derived from the World Health Organization (The WHOQOL Group, 1998a,b). The 26-item World Health Organization Quality of Life (WHOQOL) instrument uses a Likert scale and produces four quality-of-life scores: (a) physical health (e.g., “To what extent do you feel that physical pain prevents you from doing what you need to do?”), (b) psychological health (e.g., “To what extent do you feel your life to be meaningful?”), (c) social relationships (e.g., “How satisfied are you with the support you get from your friends?”), and (d) environment (e.g., “How safe do you feel in your daily life?”). With the current sample, the WHOQOL generated the following Cronbach’s alpha scores: physical health (0.75), psychological health (0.80), social relationships (0.73), and environment (0.81).

### Data Analysis

Responses downloaded from Qualtrics were entered into a database hosted by the IBM SPSS Statistics for Macintosh, version 24 (IBM Corp., Version 24.0; Armonk, NY, United States). Categorical variables were presented as absolute and relative frequencies (i.e., gender, marital status, academic standing, self-rated English proficiency), while continuous variables were represented by means and standard deviations (i.e., age, length of stay, acculturative stress, quality of life). An analysis of variance (ANOVA) assessed the differences between the means of acculturative stress among the three groups of Chinese international students (undergraduate, master’s, and doctoral students). Spearman correlations were calculated as an exploratory analysis to examine bivariate associations between variables. Controlling for sociodemographic variables, linear regression analyses were carried out to explore the associations between acculturative stress and each of the four domains of quality of life. The level of significance was set at 0.05. We deleted data that missed 70% or above of the variable (Scheffer, 2002). Drawing insights from the literature, the multiple imputation method was used to replace values that had less than 60% of the data missing (Blankers et al., 2010).

## Results

### Participant Characteristics

In total, 751 participants completed the survey among the 2,321 who received it. No differences were found between students who completed the survey and those who did not. The sample (*M*_age_ = 24.39, *SD* = 4.18) consisted of 380 women (50.7%), 271 (36.1%) undergraduate students, 213 (28.4%) master’s students, and 267 (35.6%) doctoral students. Most participants were single (*n* = 535, 71.2%). The average length of stay in the United States was 31.85 months (*SD* = 26.87). Regarding English proficiency, 334 students (44.5%) self-rated it as either average or below average, 412 (54.9%) reported it as good or very good, and 5 (0.6%) did not indicate their proficiency level (see Table 1).

**TABLE 1:** Descriptive statistics of key study variables (*N* = 751).

Variables	
**Gender, *n* (%)**	
Female	380 (50.7)
Male	370 (49.3)
Age in years, *M* (*SD*)	24.39 (4.18)
**Marital status, *n* (%)**	
Single	535 (71.2)
Non-single	216 (28.8)
**Academic standing, *n* (%)**	
College	271 (36.1)
Master’s program	213 (28.4)
Ph.D. program	267 (35.6)
Length of stay in months, *M* (*SD*)	31.85 (26.87)
**English proficiency, *n* (%)**	
Average or below average	334 (44.5)
Good or very good	412 (54.9)
Did not state	5 (0.6)
Acculturative stress, *M* (*SD*)	85.71 (23.13)
**Quality of life, *M* (*SD*)**	
Physical health	14.57 (2.34)
Psychological health	14.14 (2.31)
Social relationships	13.87 (3.02)
Environment	14.06 (2.17)

### Correlations of Key Research Variables

**FIGURE 1:**
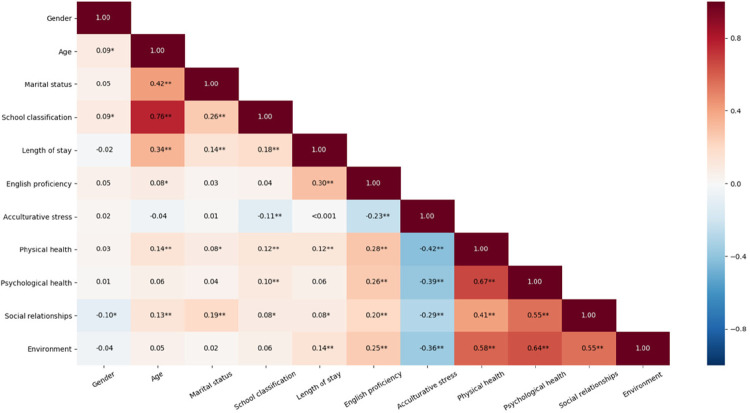
Correlations of key study variables (*N* = 751). ^∗^Red colors represent positive correlations, while blue colors denote negative correlations. The darker or more saturated the color, the stronger the relationship. Two asterisks (i.e., ^∗∗^) are used to highlight *p*-values that are less than 0.05, whereas one asterisk (i.e., ^∗^) is used to refer to less than 0.1 *p*-values.

Spearman correlations revealed significant correlations between students’ acculturative stress and physical health (*r*_*s*_ = −0.42, *p* < 0.001), psychological health (*r*_*s*_ = −0.39, *p* < 0.001), social relationships (*r*_*s*_ = −0.29, *p* < 0.001), and environment (*r*_*s*_ = −0.36, *p* < 0.001). All four quality-of-life domains were significantly intercorrelated, with *r*_*s*_ ranging from 0.55 to 0.67, *p* < 0.001 (please refer to Figure 1).

### Regression Analyses of Key Research Variables

Four multiple regression analyses were conducted to examine the variance in students’ quality of life (i.e., physical health, psychological health, social relationships, and environment) explained by demographics, length of stay in the United States, self-rated English proficiency, and acculturative stress. Findings showed that all four multiple regression analyses were significant (please see Table 2). In all analyses, self-rated English proficiency was a positive predictor of students’ physical health (β = 0.20, *p* < 0.001), psychological health (β = 0.19, *p* < 0.001), social relationships (β = 0.13, *p* < 0.01), and environment (β = 0.17, *p* < 0.001). Acculturative stress was a negative predictor of physical health (β = −0.34, *p* < 0.001), psychological health (β = −0.28, *p* < 0.001), social relationships (β = −0.23, *p* < 0.001), and environment (β = −0.28, *p* < 0.001).

**TABLE 2:** Multiple regression analyses of key research variables (*N* = 751).

Predictor variables	*B*	*SE B*	β	*t*	*p*	Adj. R^2^
**Dependent variable: Physical health QoL**						
Gender	0.17	0.18	0.04	0.96	0.34	
Age	0.07	0.03	0.12	1.93	0.05	
Marital status	0.02	0.22	0.004	0.10	0.92	
Academic standing	0.02	0.15	0.01	0.14	0.89	
Length of stay	–0.001	0.004	–0.01	–0.15	0.88	
Self-rated English proficiency	0.94	0.19	0.20	4.90	<0.001	
Acculturative stress	–0.03	0.004	–0.34	–8.59	<0.001	0.20
**Dependent variable: Psychological health QoL**						
Gender	–0.004	0.19	–0.001	–0.02	0.98	
Age	–0.01	0.04	–0.02	–0.27	0.79	
Marital status	0.11	0.23	0.02	0.49	0.63	
Academic standing	0.22	0.15	0.08	1.47	0.14	
Length of stay	–0.003	0.004	–0.03	–0.64	0.52	
Self-rated English proficiency	0.86	0.20	0.19	4.28	<0.001	
Acculturative stress	–0.03	0.004	–0.28	–6.76	<0.001	0.14
**Dependent variable: Social relationship QoL**						
Gender	–0.77	0.25	–0.13	–3.10	0.002	
Age	0.05	0.05	0.07	1.12	0.26	
Marital status	0.90	0.31	0.13	2.88	0.004	
Academic standing	–0.02	0.20	–0.004	–0.08	0.94	
Length of stay	–0.001	0.01	–0.01	–0.18	0.86	
Self-rated English proficiency	0.81	0.27	0.13	3.02	0.003	
Acculturative stress	–0.03	0.01	–0.23	–5.47	<0.001	0.12
**Dependent variable: Environmental QoL**						
Gender	–0.11	0.18	–0.03	–0.60	0.55	
Age	–0.004	0.03	–0.01	–0.11	0.92	
Marital status	0.03	0.22	0.01	0.16	0.88	
Academic standing	–0.01	0.14	–0.004	–0.06	0.95	
Length of stay	0.01	0.004	0.07	1.46	0.15	
Self-rated English proficiency	0.73	0.19	0.17	3.86	<0.001	
Acculturative stress	–0.03	0.004	–0.28	–6.63	<0.001	0.13

*Adj., adjusted; QoL, quality of life.*

Results indicated that among the three student groups, undergraduates had the highest acculturative stress mean score of 88.70 (*SD* = 24.36), followed by master’s students (*M* = 85.82, *SD* = 21.85); doctoral students had the lowest score of 82.88 (*SD* = 22.58). An ANOVA addressed the second aim (i.e., whether significant differences manifested in acculturative stress between the three groups). We observed significant differences between groups [*F*(2,688) = 3.934, *p* = 0.020]. A *post hoc* test using Tukey’s HSD revealed a significant difference in acculturative stress scores between undergraduate students and doctoral students (*p* = 0.014); however, no significant difference emerged between undergraduate and master’s students (*p* = 0.392) or between master’s and doctoral students (*p* = 0.370).

## Discussion

Acculturative stress negatively shapes the physical and psychological health and well-being of international students (Al-Krenawi et al., 2020; Dorevitch et al., 2020; Jenkins and Boyd, 2020; Liu et al., 2020; Minutillo et al., 2020), a phenomenon that could be further exacerbated by global crises such as international trade wars and the COVID-19 pandemic (Ma and Miller, 2020; Su et al., 2020; Zhai and Du, 2020; Zheng et al., 2020). However, there is a lack of research that sheds light on the intensity, impacts, and consequences of acculturative stress on international students’ health and well-being, particularly those of Chinese origin. During the 2019–2020 academic year alone, international students studying in the United States, have collectively contributed $44 billion to the economy and supported approximately 458,290 jobs during their stay – over a third (34.6%) of this student body are Chinese international students – the most significant international student group in the country (Institute of International Education, 2020). What is also worth noting is that many contributions international students made to the United States society, such as improving America’s intellectual prowess, innovative power, and cultural diversity (Chellaraj et al., 2005; No and Walsh, 2010; Ghosh et al., 2014; Sato et al., 2019), might be too invaluable to put a price tag on. In other words, research on investigating and addressing international students’ health challenges is not only urgently needed from a humanitarian perspective, but also indispensable out of social and economic concerns and considerations.

Addressing this research gap, this study investigates the interplay between international students’ academic standing, acculturative stress, and quality of life. To our knowledge, this is the first study that examined the aforementioned relationship in a large geographically representative sample of Chinese international students studying in the United States. Our first research question aims to evaluate the impact of acculturative stress on Chinese international students’ quality of life. Findings show that acculturative stress negatively affects students’ quality of life regardless of their academic standing. Data analyses further reveal that the negative impact of acculturative stress is permeated through all aspects of international students’ quality of life, ranging from physical and psychological health to social relationships.

The second research question aims to study the role of academic standing in shaping the relationship between international students’ acculturative stress and quality of life. The results show that, compared to graduate students, undergraduates shoulder greater acculturative stress, and in turn, experienced poorer quality of life. Interestingly, the relationship is only significant between doctoral and undergraduate students. In other words, statistically significant differences only emerged between undergraduate and doctoral students’ acculturative stress and quality of life, but not among undergraduate and master’s students or master’s and doctoral students. One way to shed light on these within-group differences is through a nuanced understanding of the relationship – it is possible that the differences in academic standing and experience, and by extension, students’ abilities to cope with their acculturation processes, between doctoral and undergraduate students are meaningfully more pronounced than those of doctoral and master’s students or master’s and undergraduate students. In other words, compared to undergraduates, doctoral students might have markedly advanced skills and knowledge needed to cope with American culture as well as the United States higher education system (Institute of International Education, 2020), capabilities that master’s students may not sufficiently possess. A key takeaway of this finding is that, considering that the academic experience is an adjustable variable, universities and colleges can employ their existing academic apparatus and resources to boost international students’ academic performance and lessen students’ acculturative stress and improve their quality of life.

In line with previous research (Smith and Khawaja, 2011; Lopez and Bui, 2014), self-rated English proficiency was a significant predictor of international students’ acculturation experience. Different from some studies, which suggest that length of stay in the United States significantly shape international students’ acculturative stress levels (Koo et al., 2021), the current findings indicate that the relationship was insignificant. One way to shed light on this finding is via gaining a deeper understanding of the scales studied. It is important to note that different from the length of stay, which is primarily an objective measure, self-rated English proficiency is subjective. In other words, rather than a true reflection of the students’ English proficiency, such as (most recent) English language test scores, self-rated English proficiency is more likely to reflect students’ confidence with their English capabilities, and by extension, their confidence in interacting with their cultural, social, and academic environments. To further explore these relationships, future research could investigate objective English proficiency measures (e.g. TOEFL or GRE scores), subjective English proficiency scales, length of stay, as well as acculturative stress among international students in the same research context to tease out the nuanced differences in these variables and how these variations might influence international students’ acculturation experience.

Another critical area of research that could help explain the differences in undergraduate, master’s, and doctoral international students’ experiences of acculturative stress centers on the role of perfectionism. Perfectionism could be best understood as the degree to which individuals have high goals and aspirations for themselves (Flett et al., 1989). Mounting evidence indicates that maladaptive perfectionism (i.e., discrepancies between individuals’ expectations and performance) is common among college students (Aparicio-Flores et al., 2020a,b; Vicent et al., 2020), especially international students (Huang and Mussap, 2018; Dorevitch et al., 2020; Lee et al., 2020). Research shows that international students’ acculturative stress is significantly associated with their levels of perfectionism and years in the host country (Wei et al., 2007).

However, there is a lack of research investigating the role of academic standing in this particular population. Compared to their master’s and doctoral counterparts, undergraduates might have a high level of maladaptive perfectionism which could result in greater degrees of acculturative stress. Furthering the role of perfectionism in international students’ acculturative stress, future research could examine how perfectionism dimensions might shape international students’ acculturative stress and quality of life. In addition, many other potential confounders that might affect international students’ acculturative stress, such as the students’ socioeconomic background, health, and even the characteristics of the programs they are in, should also be further investigated to gain a more in-depth understanding of international students’ acculturative stress and possible intervention strategies.

### Limitations

While this study fills critical gaps in the literature, it is not without limitations. First, the study survey was self-administered, which means that findings may be susceptible to social desirability and recall biases. A raffle of a $50 Amazon gift card was offered to show gratitude to students’ input and incentivize participation. It is possible that the incentive might result in possible unintended selection bias (e.g., students who are less interested in financial incentives might have chosen to not participate in the survey). Second, the cross-sectional nature of this work means that limited causal conclusions can be drawn from the data. It is also important to note that one item in the WHOQOL questionnaire was accidentally excluded from the survey design. While the error was addressed with the multiple computation method for missing values (Scheffer, 2002), it is a mistake, albeit unintentional, needs to be acknowledged.

There are many ways to classify academic standing, but we only looked at degree level; future research could address this limitation by investigating other aspects of international students’ academic standing and experience. To establish researcher-participant rapport, no force response or quality control measures was deployed in the survey. Though in hindsight, both measures should have been integrated into the questionnaire to boost survey robustness. Aiming to partially address these flaws as well as to gauge international students’ acculturative stress amid the coronavirus 2019 (COVID-19) pandemic, the researchers are working on developing a follow-up study that will employ both force response and quality control mechanisms in the survey design.

## Conclusion

This study is the first to explore the associations between international students’ academic standing, acculturative stress, and quality of life in a nationally representative sample of Chinese international students studying in the United States. Our study found that, compared to master’s and doctoral students, undergraduates had greater acculturative stress, which was associated with lower levels of quality of life. This finding highlights the potentially positive role of academic experience – while acculturative stress deteriorates international students’ quality of life, students’ academic standing and experience could be the protective factor in the equation. Future research could further examine how universities and colleges can capitalize on their academic apparatuses and resources to improve international students’ academic performance and students’ acculturation experience and quality of life.

## Data Availability Statement

The raw data supporting the conclusions of this article will be made available by the authors, without undue reservation.

## Ethics Statement

The studies involving human participants were reviewed and approved by the IRB protocol number: 2014-12-0027, issued by The University of Texas at Austin’s Institutional Review Board. Written informed consent for participation was not required for this study in accordance with the national legislation and the institutional requirements.

## Author Contributions

ZS developed the research idea and drafted the manuscript. DM, JW, FS, BL, XL, YC, LY, and Y-TX reviewed and revised the manuscript. All authors contributed to the article and approved the submitted version.

## Conflict of Interest

FS is an employee of Shanghai United Imaging Intelligence Co., Ltd. The company has no role in designing and performing the surveillances, and analyzing and interpreting the data. The remaining authors declare that the research was conducted in the absence of any commercial or financial relationships that could be construed as a potential conflict of interest.

## Publisher’s Note

All claims expressed in this article are solely those of the authors and do not necessarily represent those of their affiliated organizations, or those of the publisher, the editors and the reviewers. Any product that may be evaluated in this article, or claim that may be made by its manufacturer, is not guaranteed or endorsed by the publisher.
